# Epigenetic Regulation of the Mammalian Cell

**DOI:** 10.1371/journal.pone.0002290

**Published:** 2008-06-04

**Authors:** Keith Baverstock, Mauno Rönkkö

**Affiliations:** 1 Department of Environmental Science, University of Kuopio, Kuopio, Finland; 2 Department of Computer Science, University of Kuopio, Kuopio, Finland; University of Munich and Center of Integrated Protein Science, Germany

## Abstract

**Background:**

Understanding how mammalian cells are regulated epigenetically to express phenotype is a priority. The cellular phenotypic transition, induced by ionising radiation, from a normal cell to the genomic instability phenotype, where the ability to replicate the genotype accurately is compromised, illustrates important features of epigenetic regulation. Based on this phenomenon and earlier work we propose a model to describe the mammalian cell as a self assembled open system operating in an environment that includes its genotype, neighbouring cells and beyond. Phenotype is represented by high dimensional attractors, evolutionarily conditioned for stability and robustness and contingent on rules of engagement between gene products encoded in the genetic network.

**Methodology/Findings:**

We describe how this system functions and note the indeterminacy and fluidity of its internal workings which place it in the logical reasoning framework of predicative logic. We find that the hypothesis is supported by evidence from cell and molecular biology.

**Conclusions:**

Epigenetic regulation and memory are fundamentally physical, as opposed to chemical, processes and the transition to genomic instability is an important feature of mammalian cells with probable fundamental relevance to speciation and carcinogenesis. A source of evolutionarily selectable variation, in terms of the rules of engagement between gene products, is seen as more likely to have greater prominence than genetic variation in an evolutionary context. As this epigenetic variation is based on attractor states phenotypic changes are not gradual; a phenotypic transition can involve the changed contribution of several gene products in a single step.

## Introduction

Today one of the most pressing issues in biology is to understand how the epigenetic aspects of the cell are regulated, that is, how the appropriate gene products are brought into action when and only when appropriate. Writing in 1958 Nanney [1] poses, under the heading “Epigenetic Control”, the question of whether it is a “template replicating mechanism”, i.e. DNA replication, or “some other” unspecified mechanism, which manifests phenotype at the cellular level. In essence Nanney was questioning whether all the then known empirical evidence about biological function, which he reviews, regarding the stability of phenotype could be accounted for as a result of “genetic regulation”, or whether there was a need to invoke “epigenetic regulation” in addition. He concludes by nominating two separate mechanisms by which “homeostasis” could be achieved, namely a replicating template mechanism or another, “*perhaps self-regulating metabolic patterns*” as suggested by Delbrück at a Congress on Genetics in 1949. In the discussion following a paper that had attributed a specific phenomenon to the reproduction of genes that were favoured or inhibited by environmental conditions Delbrück noted that “*many systems in flux equilibrium are capable of several different equilibria under identical conditions. They can pass from one state to another under the influence of transient perturbations*.” [2] Today we would refer to “flux equilibrium” as a dynamic steady state.

In the event biology has invested heavily in the “template replicating mechanism” to the almost complete exclusion of any alternative. Prior to 1953 the concept of a gene was much more fluid than it is today being based primarily on empirical evidence of how it could be inherited and mutated. However, it can be argued that the case made by Schrödinger in 1943 [3], on quantum mechanical grounds, that the property “life” could not be based on statistical averaging, as is for example, temperature, and must therefore (because at that time there was no obvious alternative) be based on a mechanism (he used the analogy of a clock based on an aperiodic crystal) was highly influential in the subsequent development of cell and molecular biology. The extraordinary elegance of DNA as a semi-conservative replicating mechanism seems to have sealed the fate of the subject up to at least 2000.

Phenomena such as imprinting and the fact that a single genotype gives rise to more than 200 cellular phenotypes, could not, however, be explained without resort to some kind of “extra genetic” or epigenetic phenomenon. Indeed, assuming that all the information necessary to regulate the deployment of the code is encoded in the genotype leads to an infinite regression. Today there is a high degree of consensus that imprinting and other aspects epigenetic regulation are controlled by chemical marking, methylation and acetelylation, of DNA and the histones in chromatin [4], [5] and that these marks also constitute the epigenetic memory [6]. These it is generally assumed serve in a complex manner and in conjunction with sequences in the genome associated with coding regions, to regulate the transcription process. The study of the role of these “epigenetic marks” is now a major activity in cell and molecular biology [7].

In parallel and in recognition of the fact that separating the genome into fragments for detailed study followed by re-synthesis has limits as a strategy for understanding biology, approaches under the heading of “systems biology” have burgeoned. However, as is made clear by O'Malley and Dupré [8] it is far from clear what exactly the term “systems biology” means. They define two main approaches, namely pragmatic (labelled type 1) and theoretic (type 2). The majority of systems biologists are of type 1 and “*for them “system” is a convenient but vague term that covers a range of detailed interactions with specifiable functions*.” [8]. Type 2 systems biologists see a fundamental aspect to the term “system” along the lines of that advanced by Bertalanffy [9] as general systems theory. The essence of this approach is that the system is thermodynamically open and that the high level properties of a system, such as phenotype, emerge from the global *interactions* of its component parts to give a result that is greater than the sum of those parts. This leads Huang [10] to distinguish between types 1 and 2 by the terms “localist” and “globalist”.

Recently uncovered features of the cell would argue strongly for the globalist perspective as the more likely to be relevant to understanding biology. For example, detailed study of chromatin in the nucleus of eukaryotic cells has revealed substantial order in respect of both the location of gene coding sequences and of discrete chromosome territories within the “nuclear architecture” that are associated with gene regulation [11]–[15]; indeed, Fraser and Bickmore [15] conclude that “*the genome's spatial organisation is a key contributor to function.*” In a comparison of the differentiation of haemopoietic cells to neutrophils and erythroid cells it was found that co-regulated gene sequences were clustered in chromosomes and spatially proximal in the nucleus [16]. These results suggest that epigenetic regulation is indeed a global genomic phenomenon involving both spatial and conformational transitions in chromatin among other features.

Here we propose a hypothesis/model based on recognised features of the cell to describe the epigenetic regulation of the mammalian cell as a system somewhat similar to the concept Delbrück advanced in 1949 [2], namely based on dynamic steady states and thermodynamically open. We strive for realism in our assumptions recognising that the complexity of the model may make it computationally relatively intractable. However, we believe that the qualitative understanding of the way the cell operates would provide the most relaible basis for simplifying the model. We examine the evidence that supports the model and discuss its implications for understanding the processes that regulate cells.

We start with the phenomenon of genomic instability as induced by ionising radiation [17]. Previously we have drawn attention to the implications of the chemically friable nature of DNA under physiological conditions [18] and subsequently described genomic instability as a stochastic epigenetic phenotypic transition between attractors, essentially specific patterns of gene products active in the cell, representing phenotype [19]. Subsequently, Huang et al [20] have identified, experimentally, such attractors as representatives of phenotype in the chemically induced differentiation of neutrophil precursors to the terminally differentiated state. Essentially, the chemical perturbation of the precursor attractor stimulates the transition to other attractors [21] and ultimately the terminally differentiated state.

Attractors are components of a state space with a dimension for each of the gene products coded for by the genotype, i.e., more than 100,000 in the human. A typical attractor might involve between 1000 and 10,000 active gene products. The attractors within the system are defined by rules of engagement between gene products and envisaged to be essentially point attractors, as opposed to limit cycle attractors, but they should be seen as elements of a limit cycle representing the cell cycle.

In the case of radiation induced genomic instability the physical properties of energy deposition by ionising radiation and the low doses required to initiate genomic instability indicate that the transition to instability is not a genetic effect and must therefore be epigenetic in character [22], [23]. It has been proposed [19] that the normally stable phenotype of a cell is represented by an evolutionarily conditioned or “home” attractor, that is, one that has been evolutionarily selected most importantly for two properties, namely robustness, or resistance to perturbation and fidelity in the replication of the genotype, or stability.

**Figure 1:**
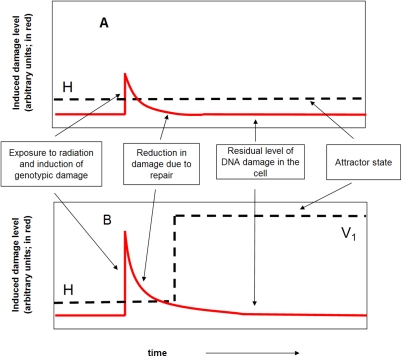
Illustration of responses of the system to genotypic damage. Panel A: A relatively small exposure to ionising radiation creates damage in the genomic DNA (red trace) which is detected and repaired by the cell. The prevailing home attractor, H, is perturbed but not irreversibly so, i.e. the basin of attraction is not exceeded and the system stays in the H attractor. Panel B: A larger exposure to ionising radiation but still within the capacity of the cell to repair causes the cell to exceed the basin of attraction of the home attractor, H, and stimulates the irreversible transition to the variant attractor V_1_.

Exposure to ionising radiation, because it causes molecular damage to the genomic DNA and therefore genotype, which, if not repaired prior to cell division may compromise the genotype, thus places increased demands on the on going damage detection and repair processes in the cell, which are components of the home attractor. If that stress exceeds a critical value an irreversible, due to the high dimensionality of the attractor, transition to a variant and unconditioned attractor is stimulated. See Figure 1. If the cell can survive and divide at the variant attractor it will accrue the genotypic damage that characterises the instability phenotype by virtue of a lower level of fidelity in replication. The genomic instability phenotype is thus a mutator phenotype.

Thus, genomic instability can be seen as the *loss,* at the cellular level, of the ability to replicate the genotype with the optimal level of integrity that was *gained* substantially through evolutionary conditioning subsequent to the origin of the species to which that cell belongs. In effect evolutionary conditioning minimises the residual damage in the dynamic steady state between DNA degradation and repair; the conditioning is thus a purely epigenetic evolutionary selection process.

**Figure 2:**
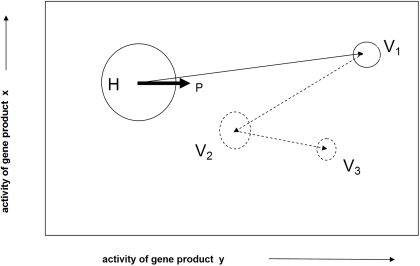
Illustration of a state space. The figure illustrates a very simplified state space for a two dimension system, the coordinates indicating the activities of gene products x and y. The potential attractors are represented by circles, the diameters of which are proportional to their basins of attraction, the home attractor H being the largest because of environmental conditioning. A perturbation P of H beyond the basin of attraction due to an increase in gene product y causes the adoption of variant attractor V_1_. This is the initiation step of genomic instability. Subsequently, due to the relatively reduced robustness of variant, i.e., unconditioned, attractors, further transitions (dotted lines) to other variant attractors characterises the genomic instability phenotype.

It is the openness of the cell to its environment that is at the root of the instability phenomenon. Exposure to radiation, an extrinsic agent, causes the cell to respond to detect and repair the damage to the genotype. The consequent increased demand for the gene products responsible for detection and repair of the damage represents a perturbation of the attractor, which if sufficiently severe will exceed the basin of attraction in respect of one or more gene products and thus the adoption of the variant attractor. (See Figure 2)

## Methods

### Statement of hypothesis and definition of terms

**Figure 3:**
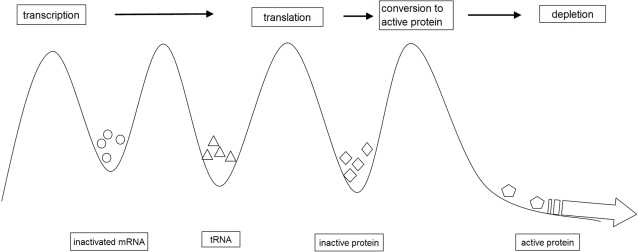
Illustration of the multi-compartmental dynamic steady state. The figure illustrates the multi-compartmental dynamic steady state initiated by the transcription of coding sequences to mRNA, which is translated to peptide and finally yields active gene products which are depleted (block arrow) through use. There may be additional depletion (not indicated) by spontaneous decay from the product compartments.

Our hypothesis is that the cellular phenotype of a mammalian cell is represented by a complex high dimensional dynamic attractor embedded in a state space with a dimension for each active gene product encoded in the genotype. The state space is therefore a proteomic state space. The active gene products are assumed to interact selectively through rules of engagement, which are non-deterministic to allow for interactions with the environment, including other cells in the organism (one aspect of the openness). We further assume that the gene products are metabolised by the system (a second aspect of openness) and we assume there exists for each gene product a multi-compartmental dynamic steady state originating in the transcription of the coding sequences and terminating in the depletion of the active gene product, either as a result of it having been incorporated into the cellular architecture or selectively destroyed after use or being subject to spontaneous degradation. See Figure 3. This is referred to as the post-transcriptional dynamic steady state. Prior to use gene products are stored or present in inactive forms, mRNA, tRNA, unfolded peptide and inactive protein. Being an open system driven by attractors there is no continuum of stable states in the system; stable states are “quantised” at the discrete high dimensional attractors.

The attractors available for occupation in the state space are an emergent property of the system determined by the rules of engagement, which also give rise to an architecture that influences transitions between attractors. The rules of engagement can be seen as the edges in a network, the nodes of which are the gene products and as does the genotype, they exhibit selectable variation. It is therefore assumed that they have been acted upon in evolutionary terms to increase the fitness of the architecture, including the attractor locations in the state space. Attractor transitions are equivalent to phenotypic transitions and thus represent biological processes at the cellular level such as differentiation, carcinogenesis and evolution.

The system: It is important to be clear about the boundary between the system and its environment. In this case we define the system as the mammalian cell and all the material therein. However, we exclude the informational content (base sequence) of the genomic DNA but not the substance. Thus, the system is open in the sense that coding information derives along with other non-system “information” from the environment, including the neighbouring cells in the tissue and organism as well as, where appropriate, cohabiting organisms [24] such as bacterial flora, and the environment beyond, for example, ionising radiation and chemicals.

Gene products: these are the proteins and certain of the RNA species, specifically the microRNAs, manufactured in the cell and which either are incorporated into the cellular structure or used by the system. We are interested in the behaviour of these gene products with time. We denote time by **t**. Consider a specific gene product, **gp**. Then, the activity **m** of the gene product at any given time is captured as a function of time, **m_gp_(t)**.

Attractor: Attractors are an emergent property of the system, which occupy a “point” or “volume” of the state space and are surrounded by a basin of attraction from which states drain into the attractor. It thus represents a domain of stability, albeit, limited by the boundary of the basin of attraction. Each gene product **gp** is governed by an attractor **a_gp_**. The attractor determines a value range, a lower and an upper bound for the activity of the gene product. We denote the range of the attractor by **[low_gp_,up_gp_]**. In particular, if the activity of the gene product is within the attractor range, it remains there. In other words, if **low_gp_≤m_gp_(t1)≤up_gp_** holds for some **t1**, then also the condition **low_gp_≤m_gp_(t2)≤up_gp_** holds for any **t1<t2**. Each attractor **a_gp_** determines a basin **b_gp_**. The basin of the attractor is a value range, indicating the minimum and maximum bounds for the activity of the gene product, such that if the activity of the gene product is within the range, it will eventually reach a value within the attractor range. We denote the range of the basin by **[min_gp_,max_gp_]**. Thus, formally, if **min_gp_≤m_gp_(t1)≤max_gp_** holds for some **t1**, then there exists also **t2** such that **t1<t2** and **low_gp_≤m_gp_(t2)≤up_gp_** holds. In addition to the attractors and their basins of attraction, there exist volumes of state space through which the system transits during transitions and which exert some influence over the direction of migration.

Dynamic steady state: a condition in which two or more opposing processes are balanced to produce a stable state. Two categories are of particular interest, namely the DNA degradation under physiological conditions (due to, for example, hydrolysis) opposed to the repair of that degradation by cellular repair processes, and the metabolic process generating gene products, commencing with their transcription opposed to their depletion through use, the post-translational steady state. (See Figure 3)

Rule of engagement: The rules of engagement speak of the active gene products in time. Consider, for instance gene products **gp_a_** and **gp_b_**. Then a rule is of the generic form *“IF* ***gp_a_*** *is active THEN* ***gp_b_*** *is active”*, stating that the activity of **gp_a_** *implies* the activity of **gp_b_**. Formally, the activity of a gene product is expressed with respect to some activity ranges, **r_gpa_** and **r_gpb_**, at points **t1** and **t2** in time. Then, a rule of engagement is a relation: **m_gpa_(t1)∈r_gpa_⇒m_gpb_(t2)∈r_gpb_**. For a gene product there are typically many rules of engagement and, thus, a gene product can be engaged with several other gene products. Consequently, a perturbation of any one gene product has the potential to perturb all those with which it is engaged.

Stability: within this context stability refers to the ability of the genome to replicate its genotype with maximum fidelity. DNA is an unstable compound under physiological conditions and thus is subject to ongoing repair. Degradation and repair are opposing processes which create a dynamic steady state of minimal residual damage in the system at any point in time [18], [19]. This dynamic steady state is crucial for the long term stability of the system.

Robustness: the property of the system to resist perturbations of its stable states and of its transitions between stable states.

Homeostasis: is the property of an open system to regulate its internal state and maintain a stable condition.

Evolutionary conditioning: an evolutionary process whereby variations in the rules of engagement are selected particularly if they improve the integrity of the replication of the genotype, i.e. enhance stability, and/or expand the basin of attraction of the attractor, thus enhancing robustness.

## Results

### Description of the operation of the system

The system comprises two primary components, namely, the rules of engagement governing the regulation or deployment of the gene products and the material which is regulated to “build” the system. Placed in the environment, rather than the system, is the genotypic information that codes for the gene products. The reason for this is that the rules of engagement can be regarded as the formal causal component of the system (the syntax). The residue, genomic coding sequence and environmental influences, are then regarded as the semantic component. Separating them in this way, as does for example Rosen [25], [26], allows for a clearer logical definition [8] and treatment of the system.

Spatial distribution of gene coding sequences in the nucleus ensures that the gene products that are required by the current attractor are available to be drawn upon [15], [16]. It is assumed that the gene coding sequences are transcribed stochastically as and when two conditions are met, namely that the chromatin structure is appropriate for the transcriptional apparatus to access the coding sequence and the sequence is activated for transcription. The transcribed products are stored (usually) in inactive forms in multi-compartmented dynamic steady states (one for each gene product) as a component of the routine metabolic activity of the cell. See Figure 3.

In general, if an attractor **a_gp_** is perturbed, i.e., the value for **m_gp_(t)<min_gp_(t)** or **m_gp_(t)**>**max_gp_(t)** for one or more gene products, the system will exit the current attractor and adopt a variant attractor **v_gp_**.

Two circumstances in which the prevailing attractor can be perturbed are now considered. One can be regarded as *scheduled* within the system and its environment and thus part of its normal operation and the other *unscheduled* or stochastic and “forced” from the environment.

Differentiation is the most common scheduled phenotypic transition between attractors at the cellular level. It can be initiated through signalling from its close environment or from within the system. It can be induced in the laboratory by specific drugs [20]. There are several ways of perturbing the existing state of the attractor. For example, a change in the level of activity of specific gene products can be induced by acting on the inactive protein of a specific gene product precursor to up-regulate the gene product, or by transfer of active gene product from the cytoplasm to the nucleus. In effect any perturbation of the activity of a gene product, **m_gp_**, up- or down-regulation, that places it outside the range of activities of the attractor and its basin, **m_gp_(t)**<**min_gp_**, or **m_gp_(t)**>**max_gp_** will lead to a transition.

An example of an unscheduled phenotypic transition is the induction of genomic instability by ionising radiation. Here it is envisaged that stress on the post-transcriptional steady states of gene products dealing with the damage detection and repair of the genotype can cause the basin of attraction to be exceeded and the system to be released from its “normal” or “home” attractor. In this case the system will migrate to a variant attractor, which because it has not been occupied before has not been evolutionarily conditioned. It is thus likely to be less robust, i.e., a smaller basin of attraction and less stable, i.e., less proficient at error free replication of the genotype, than the normal attractor. A consequence of the loss of robustness will be that the variant attractor will be more prone to environmental perturbation and thus prone to migrate to other variant attractors. See Figure 2. For this reason the instability phenotype is best referred to an *incomplete* phenotype. A second consequence will be due to the loss of stability resulting in a mutator phenotype.

### Support for the hypothesis

We briefly review here the evidence that supports the idea that cellular regulation, both in mammalian cells and their evolutionary precursors, micro-organisms, is essentially a physical process involving transitions between dynamic attractors, which are a product of self-organisation.

That randomly organised systems can exhibit self-organisation has been conclusively demonstrated by Kauffmann [27]. Random Boolean networks, where any node connects through rules of engagement with two others on average, exhibit state cycle attractors, that is, as the system is refreshed by applying the rules sequentially and repeatedly to each node in turn, the system settles into a relatively short cyclic sequences of states, a state cycle attractor. Naturally evolved networks, including genetic networks, tend to be of the scale free rather than random [28] and these also exhibit robust self organisation [29].

Studies of micro-organisms from which it is assumed that multi-cellular organisms evolved demonstrate the ability of cells subject to environmental stress to adapt to previously un-encountered conditions. Most notably the experiments of Kashiwagi et al [30] show that a micro-organism with a synthetic bi-stable gene switch that is able to exploit two nutritional environments but with mutual inhibition (so that both do not operate simultaneously) can adopt an adaptive attractor state that is able to exploit the alternative nutrient if deprived of the prevailing nutrient. In other words the organism switches from one state to the other according the availability of nutrient and since the apparatus that enables the switch is artificial there can be no existing signalling transduction pathway for it. Bacteria, unlike mammalian cells, continuously transcribe their gene products directly to the active state and attractors form spontaneously.

Kashiwagi et al [30] argue that the range of potential environmental stresses to which cells are exposed must be much larger than the signal transduction pathways that have evolved to meet such challenges. Thus, cells must have the ability to select adaptive attractors in the absence of any evolved process. They propose that this property is a general consequence of the stochastic nature of the network dynamics. In the absence of nutrient cellular activity falls and the stochastic process of transcription generates transcriptional noise. If as a result an adaptive attractor is encountered, allowing a higher cellular activity and thus turnover of mRNA and production of appropriate gene products, this suppresses the influence of the noise and the new attractor is established. These observations illustrate the fundamental nature of dynamic attractors representing phenotype in cells.

In fission yeast (*S. Pombe*) a model based on a Boolean network predicts the known sequence of activities of the gene products through the cell cycle purely on the basis of the observed biochemical interactions (rules of engagement) [31]. The model exhibits a stationary state (attractor) at the G1 stage (cell growth phase) of the cell cycle. If a single randomly chosen gene product is perturbed during the cell cycle the system reverts to the G1 attractor in the majority (81%) of the trials. Similarly in another yeast model, *S. cerevisiae*, [32] it was shown that for 2048 initial states of a network with 11 elements there were 7 fixed point attractors with 86% of final states in the attractor associated with the G1 stage of the cell cycle.

In single celled organisms transcription of the coding sequence and regulation are more-or-less synonymous (although fission yeast is an exception). For multi-celled organisms, cooperating to form a tissue or organism, a more complex form of regulation is required. We therefore postulate that before multi-cellular growth could be established measures had to evolve to regulate the production of gene products much more closely and reduce the noise at the gene product level. This we propose is achieved through the development of post-translational processes, which serve to partly de-couple regulation from transcription. Transcription is stochastic but a post transcriptional steady state in which gene products in inactive forms are stored intervenes between transcription and regulation. (See Figure 3)

Huang et al [20] have provided the first experimental demonstration in mammalian cells showing that the drug induced *in vitro* differentiation of a neutrophil precursor to the terminally differentiated state involved the transition between two high dimensional attractors representing phenotype. Human promyelocytic HL60 cells *in vitro* can be reliably stimulated to differentiate to stable neutrophils with drugs, for example DMSO. Serial measurements of gene profiling as a surrogate for the genomic state during the process induced by two drugs, showed that the differentiation pathways were dependent on the identity of the initiating drug. Thus, the concept of a single encoded differentiation pathway within the system was rejected. When the differentiation process is reversed by manipulating the drug concentration hysteresis was observed [21]. This is interpreted by the authors as indicating the presence in the differentiation process of attractor states intermediate between the precursor and the terminally differentiated state. These experiments provide strong support for the concept of cellular processes being in essence transitions between attractors representing phenotypic states, the actual “route” of the transition being dependent on the conditions initiating the transition rather than an encoded pathway.

The proposal that the well established phenomenon of genomic instability induced by ionising radiation can be understood in terms of an epigenetic transition between dynamic attractors representing phenotype was advanced in 2000 [19]. A prediction of the proposal is that once destabilised the genome will “wander” in the state space adopting variant attractors, Figure 2, and thus a destabilised clone will exhibit an increasing diversity of gene expression with time. A study of the transcription products of fresh human cells rendered unstable with ionising radiation and followed over several generations demonstrated the predicted increase in diversity of gene expression compared to unirradiated cells [33]. Furthermore, clones expanded from a single cell (4 irradiated and 4 controls) and cultured for between 22 and 46 days showed that about 43% of the transcripts were common to the irradiated and unirradiated clones. Using a variation filter the 4 clones derived from the irradiated cells showed consistently higher variation than the clones derived from the unirradiated cells. In a pair wise comparison of irradiated with irradiated and unirradiated with unirradiated clones, in only one of the 12 comparisons was the number of changed clones in the irradiated comparison less than the highest in the unirradiated comparison [33].

Thus, there is clear support for the contention that dynamic attractors represent phenotype in mammalian cells and that this has been inherited from more primitive organisms. Attractors are robust to disruptive environmental influences to a degree but beyond a limit defined by the basin of attraction, which is a product of evolutionary conditioning, the system can be irreversibly perturbed adopting the instability phenotype. This we argue is a fundamentally important property of the epigenetic regulatory system that in germ cells plays a pivotal role in evolution and in somatic cells in carcinogenesis.

## Discussion

### Implications of the hypothesis

We now describe the principal implications entailed by the hypothesis/model:

- Epigenetic regulation and epigenetic memory are fundamentally physical processes deriving in part from the intrinsic rules of engagement between active gene products and in part from extrinsic influences. At mitosis, and fusion in germ cells, the attractor is inherited to determine the phenotype of the offspring.
- Due to the influences from the environment the rules of engagement are indeterminate. Further, due to its openness the system operates far from equilibrium. This results in indeterminacy in the identities of the gene products. To deal with this inherent indeterminacy it is proposed that predicate logic systems, such as Refinement Calculus, are the most appropriate computational tools.
- Epigenetic variation exists in the form of attractors that are dormant (variant attractors) in the system but which can be occupied if the system is subject to an unscheduled expulsion from its normal attractor. When such a transition occurs there is a step change in phenotype, i.e., the change is not gradual. Epigenetic variation in germ cells could play a role in speciation. In somatic cells the adoption of a variant attractor could be the initiation of carcinogenesis.

Each of these implications will be addressed in outline here and in more detail elsewhere.

### Epigenetic regulation and memory

Epigenetic regulation can be seen as a physical process preceded by the stochastic transcription of the appropriate coding sequences, dependent on the spatial ordering of the chromatin and the “status” of those sequences and contingent on the availability of the gene product precursors contained in multi-compartment post-translational steady states. Thus, although the transcriptome reflects the regulatory processes of the cell it is not as direct a reflection as the active proteome due to the buffering effect of the post-transcriptional steady states. For example, within a minute or two of damage being inflicted on the genotype by ionising radiation H2AX labelling occurs at damage sites, checkpoints are instigated to delay replication and macroscopically discernable foci of proteins assemble around the break [34]–[36] but it is not until tens of minutes later that the system responds with transcriptional responses [37].

The epigenetic memory at mitosis involves the inheritance of the attractor by the offspring cells and thus is again a physical process. Following meiosis and fusion in germ cells the situation, specifically for male cells, is more complicated [38]–[41]. In the final stages of spermatogenesis the last traces of cytoplasm are expelled from the sperm thus precluding translation of transcripts to peptides, i.e. in effect interrupting the post translational processes. However, in principle the attractor could be sustained by the previously translated but inactive and stored precursors to gene products. It would seem reasonable to assume that attractors with low metabolic activity could thus survive the final stage of spermiogenesis through to fusion.

There is ample evidence of epigenetic inheritance of genomic instability along the germ line and the subject has been extensively reviewed recently [42], [43] so it will not be repeated here. It is important to recognise that the epigenetic inheritance of the GI phenotype is not Lamarckian in character in so far as it is wholly without direction; the GI phenotype is a purely stochastic response to an environmental stimulus.

The current view, namely that epigenetic regulation is based on chromatin and DNA marking, certainly applies to, for example, imprinting. However, marking regulates at the transcription stage and it is evident that other more “immediate” processes are involved in the second by second regulation of the system. We therefore conclude that regulation is primarily a physical property of the attractor of which marking may be a consequence. Much the same argument applies to the epigenetic memory.

### Indeterminacy

We predict that the operation of the system will be characterised by indeterminacy and thus there are implications for the computational approaches that appropriately address the system. As the system is open in respect of mass and energy flux it is far from equilibrium. Specific protein structures derived from a given peptide sequence result from the folding of the peptide and the characteristic structure is usually taken to be that with the lowest energy, i.e., the equilibrium structure. In the open environment of the cell such a restriction would not apply and many folded proteins could result from a single peptide, i.e., coding sequence. In addition many proteins have indeterminate structures [44], [45] and in some cases can adopt a binding structure under the influence of the binding site [46].

Thus, any computational approach has to be top-down and able to accommodate the inherent uncertainties. We suggest that Refinement Calculus will find an application here. Refinement Calculus [47] is a lattice-theoretic framework for reasoning. It was originally introduced as a tool for proving properties about specifications and computer algorithms, to be able to refine them into executable computer programs in a provably correct, stepwise manner. Because of this, Refinement Calculus is particularly suited for reasoning about open and complex systems, when there is only partial information available in the presence of non-determinism.

Because of its strong uniform formal foundation, built upon lattice theory and higher-order logic, Refinement Calculus bridges the gap between many popular reasoning styles, including agent based reasoning, contract based reasoning, and use of game theory. In other words, Refinement Calculus is at its best in reasoning about the precondition for reaching a certain state, when the interaction mechanisms are known only to some degree of certainty. Such a piece of information is crucial, if we wish to ensure that a set of specifications and claims about the system are consistent.

When considering a dynamically based system, the rules of engagement are seen as (partial) specifications in terms of Refinement Calculus. By measuring some of the attractors and the attractor ranges, we can then start proving the consistency of the rules, and infer other potential rules of engagement governing the system. It should be noted that due to the openness and complexity of the underlying system, the cell dynamics, there is very little hope of obtaining an algorithm-like, mechanical description of its functionality; rather, the system will most likely be described as a network of partial specifications, or rules of engagement, interacting in a non-deterministic manner. Then, Refinement Calculus provides a valuable tool, fixed-point reasoning [48], for understanding the potential outcome of those interactions. In particular, Refinement Calculus excels at finding out the governing state for some specific state to be reached by the network of rules of engagement.

However, there are indications that some measure of simplification can still result in meaningful models. For example, treating the fission yeast cell cycle as consisting of some 15 elements (gene products) operating in a Boolean fashion, i.e., either “on” or “off”, with rules of engagement in terms of either activation or suppression, Davidich and Bornholdt [31] are able derive a model that predicts the cell cycle sequence. *S. pombe* has some 4800 open reading frames so their model uses only about 0.3% of the potential dimensions of its state space. In a similar earlier study [32] on the cell cycle of *S. cerevisiae* a good model of the cell cycle was obtained using about 1.3% of the some 800 regulatory elements known to be deployed in the cell cycle.

Another potentially simplifying feature with respect to computation might be modularity [49] of the state space. By defining the cell as the “system” and all outside it as the “environment” we have recognised the relative independence of the cell as a module within the organism. Within the cell specific cell lineages consist of chains of attractors linked by scheduled transitions (but not defined pathways [20]) and thus it might be assumed that there are “barriers” that essentially isolate these “lineage domains” to some degree from the rest of the state space, enabling their treatment in relative isolation of the remainder of the cell.

We conclude that type 2 systems approaches will be productive but that indeterminacy will frustrate type 1 approaches. Indeed, even in a very limited and “idealised” network of only four genes, the “reverse engineering” from data on transcription products to infer the underlying regulatory network structure is plagued by indeterminacy [50].

### Epigenetic variation

Variant attractors are a source of evolutionarily selectable variation in addition to genetic variation. The induction of genomic instability, that is, the adoption of a variant attractor, is the adoption by the cell of an epigenetic variant. If such a transition takes place in the germ cells of an established species we can envisage two consequences relative to the originating cell. Firstly, the integrity of replication will be relatively impaired and the variant will exhibit an increased mutation rate. Secondly, the robustness of the variant attractor is likely to be reduced leading to a greater propensity to adopt further variant attractors in response to perturbations.

The first of these is self-evident; genomic instability is characterised by the accretion of damage to the genotype; it is a mutator phenotype. The reduced robustness is less obvious. In the Boolean model of fission yeast [31] the attractor size predicted by the specific network is compared with that predicted by randomly connected networks with the same number of inhibiting and activating links, self-degrading and self-activating links and the same activation thresholds. The random networks typically had smaller attractors indicating that the network specific to *S. pombe,* i.e., the one that had been subject to evolutionary conditioning, had been optimised for dynamical robustness. This would imply that a variant attractor of the fission yeast, where a random change to a rule of engagement was applied, would likely show reduced robustness.

An important feature of the adoption of epigenetic variants is that phenotypic change will not be gradual: the adoption of a variant attractor could involve a change in the contribution of several gene products in a single transition. This has implications for the evolutionary selection of epigenetic variation. Gradualism is universally accepted as fundamental to Darwinian theory [51], [52]. According to Gould the term is a “*deductive intellectual consequence of asserting that natural selection acts as the creative mechanism of evolutionary change*”. It has three meanings in the theory, namely as a means of distinguishing the theory from other so called theories such as Lamarckianism, as a means of refuting saltationism, which it is argued would compete with natural selection as the creative force behind evolution and finally supporting the view that the demonstrable micro-evolutionary process (adaptation) that is central to Darwinism, would over geological timescales produce the full diversity of life that is observed today and in the fossil record. The theory of punctuated equilibrium [53] refutes this last meaning of gradualism, requiring that the process of evolution occurs in rapid spurts followed by long periods of “equilibrium” where no or very little, change takes place, as the fossil record indicates.

It should be noted that the non-gradualism we are proposing, saltationism, does not challenge Darwin's “creative force” as the change it produces is subject to natural selection.

Depending on the specific circumstances, that is, relative loss of stability and robustness, and the extent of phenotypic change, such transitions to genomic instability in germ cells could co-evolve genetically and epigenetically, potentially resulting in evolutionary consequences ranging from minor adaptation to the origin of a new species. The initial stages in the case of speciation would be characterised by increased frequency of mutation, which over several generations would decline as integrity of replication increased and the new home attractor increased in robustness, both features that would be subject to selection for fitness.

Out of the two sources of variation it would seem that the epigenetic variation would make the more important contribution to a new species or the evolution of a new phenotypic feature, this by virtue of the non-gradual element inherent in this process. Consider the similarities in genotypes of mammalian species and the concurrent diversity in phenotypes. For example, mice have about the same number of protein coding genes as humans and over 90% of the mouse and human genomes can be partitioned into corresponding regions of conserved synteny, that is, the order of genes has been conserved since the two species diverged from a common ancestor [54]. More than 99% of the proteins in the mouse genome are shared with other mammals and 98% with humans. Similarly the chimpanzee has a genome that differs from the human genome in only 4% of the bases overall and less than 1% in gene sequences coding for proteins [55].

An overwhelmingly large fraction of the phenotypic differences between mammalian species relies on the arrangement, including scale, of a more or less common set of cellular phenotypes. Thus, in theory one could contemplate identical genotypes for mouse and man with only the rules of engagement defining the phenotypic differences.

The notion that non-gradualism underlies speciation has been discussed since Darwin's time. For example, as noted by Patrick Bateson [56], Galton used the analogy of a “rough stone” with many facets that could, if sufficiently perturbed, make a jerky transition from resting on one facet to resting on another. This analogy captures the essence of the present model.

If the transition to genomic instability takes place in a somatic cell we suggest that the end result may be malignancy. Carcinogenesis, like genomic instability, is characterised by a mutator phenotype [57]. The relative loss of stability and robustness of the instability phenotype may result in changes in epigenetic regulation and the acquisition of mutations that a) give a selective growth advantage by, for example, the loss of a checkpoint and b) preclude the complete reversal of the process by modification of the state space due to the loss of or gain of dimensions (active gene products). Again a co-evolution of genetic and epigenetic variation may result in the instability phenotype resolving into a malignant phenotype. That there is an epigenetic component to carcinogenesis has been long recognised. Early experiments transplanting malignant cells into blastocysts demonstrated that the malignant phenotype could be reversed [58], [59]. Later, malignant nuclei from mice transplanted into enucleated eggs were grown into normal embryos [60]. The view of carcinogenesis advanced here (see also [19]), while recognising the importance of mutations in achieving the “hallmarks of cancer” [61], e.g., loss of senescence, anchorage free growth, etc. sees such mutations as the consequence of an underlying and more fundamental epigenetic process that leads the system into a specific domain of the state space associated with malignancy, via a series of randomly adopted variant attractors, Figure 2. Thus, as is observed, the malignant phenotype is not well defined either in terms of the attractor that represents it or in terms of the mutations that it has acquired, although both may be “characteristic” of the disease.

### Conclusion

In his Spinoza Lectures, the philosopher John Dupré [62] says “scientific modelling is not like building a scale model of a ship ….. rather scientific models are successful to the extent that they identify the factors, or variables, that really matter”. Regarding the cell as a material system driven by external “forces” in terms of the genotype, signals from neighbouring cells in the same organism and influences from the wider environment, including in some cases other organisms, is an attempt to extract those factors. Necessarily the detail that characterises the internal working of the cell, which is the subject of mainstream cell and molecular biology, is ignored. Walter Elsasser in 1981 [63] sought principles, consistent with quantum mechanics, governing biology, where replicates at any level, organisms within a species to cells in a tissue, were characterised by intrinsic variability, and thus at odds with the concept of “mechanisms”. He concluded biology relied on selection from a vast number of states and that [hereditary] reproduction rather than being duplication (possibly with errors) was better represented as “creativity with constraints”, a process “released” by genes as operators or predicates. It is our contention that the evidence that can be garnered from the products of cell and molecular biology research since 1981 fully support Elsasser's prognosis.
